# Peak torque angle of anterior cruciate ligament-reconstructed knee flexor muscles in patients with semitendinosus and gracilis autograft is shifted towards extension regardless of the postoperative duration of supervised physiotherapy

**DOI:** 10.1371/journal.pone.0211825

**Published:** 2019-02-05

**Authors:** Aleksandra Królikowska, Paweł Reichert, Andrzej Czamara, Katarzyna Krzemińska

**Affiliations:** 1 College of Physiotherapy in Wroclaw, Wroclaw, Poland; 2 Division of Sports Medicine, Department of Physiotherapy, Faculty of Health Sciences, Wroclaw Medical University, Wroclaw, Poland; 3 Centre of Rehabilitation and Medical Education, Wroclaw, Poland; Universita degli Studi di Roma 'Foro Italico', ITALY

## Abstract

**Background:**

The observational cohort study investigated whether the flexor muscles peak torque (PT) angle shifting towards extension observed in the involved knee in patients after anterior cruciate ligament reconstruction (ACLR) using semitendinosus and gracilis tendon (STGR) autograft is associated with the postoperative physiotherapy supervision duration.

**Methods:**

From 230 ACL-reconstructed males, we identified patients after ACLR utilizing STGR autograft and divided them into those who completed supervised physiotherapy <6 months (Group I; *n* = 77) and those who completed supervised physiotherapy ≥6 months (Group II; *n* = 66). The mean follow-up time was 6.84 ± 1.47 months. The ACL-reconstructed patients were compared to 98 controls (Group III). Bilateral knee flexor muscle PT measurements were performed. The relative PT at 180°/s (RPT), PT angle at 180°/s, and range of motion at 180°/s were analysed. The RPT limb symmetry index (LSI) was calculated. Tests for dependent samples, one-way analysis of variance, post hoc test, and linear Pearson’s correlation coefficient (*r*) calculations were performed.

**Results:**

The shift towards extension was noted when comparing the ACL-reconstructed limb to the uninvolved limb (Group I, *p* ≤ 0.001; Group II, *p* ≤ 0.001) and to Group III (*p* ≤ 0.001), but it was not correlated with physiotherapy supervision duration (*r* = -0.037, *p* = 0.662). In ACL-reconstructed patients, there was a moderate association of supervision duration and knee flexor LSI (*r* = 0.587, *p* < 0.001).

**Conclusions:**

The ACL-reconstructed knee flexors PT angle shift towards extension was observed regardless of the duration of postoperative physiotherapy supervision. However, the analysis revealed that the duration of supervised physiotherapy positively influenced the RPT and LSI in patients after the ACLR.

## Introduction

The graft choice options for anterior cruciate ligament (ACL) consist of autogenous, allogenous, and synthetic grafts [1].The ipsilateral bone-patellar tendon-bone or the hamstring tendons are the most common autografts [1]. The hamstring tendons autografts include the quadruple strand semitendinosus (ST) or double strand semitendinosus-gracilis (STGR). However, there have been concerns regarding ACL-reconstructed knee flexor muscles weakness, especially when both ST and GR tendons are harvested [2]. In general, studies assessing the strength of the knee flexor muscles in patients after ACLR using hamstring tendon autografts can be divided into those assessing peak torque (PT) under isokinetic conditions [3–10], the maximal isometric torque under static conditions [6, 10], and studies evaluating maximum standing knee flexion angle [3, 5, 10]. In regards to isokinetic testing, the PT, defined as the single highest torque output produced by a muscle contraction as the limb moves through the range of motion [11], has tended to be the most commonly utilized strength variable in studies evaluating knee flexor strength in patients after ACLR. Nevertheless, the assessment of this parameter may be insufficient, as there are also authors who have observed that the ACL-reconstructed knee flexor PT angle, also called the angle of occurrence referring to the angle at which PT occurs in the ROM, is shifted towards extension in patients after ACLR using a hamstring graft [12]. According to the literature, the knee flexor muscles weakness observed in deeper angles of knee flexion angles may influence performance in sports, in which knee flexion strength is required at deep flexion angles, such as in gymnastics, judo or wrestling [3, 5, 13]. Although no studies have examined the effect of post-harvesting hamstring weakness in the population of athletes practising these particular disciplines, the issue may seem important in making decisions about the type of graft to use.

Previous studies have shown that postoperative physiotherapy lasting for six months under the direct supervision of a physiotherapist favourably affects involved and uninvolved knees flexor muscles PT in males after ACLR with the use of ipsilateral STGR tendons autograft [14]. Taking into account the goals and specificity of the rehabilitation stages preparing the patient to return to physical activity at the level of recreational and competitive sport [14–16], it seems interesting whether shortening the duration of physiotherapist's direct supervision over the therapeutic process may affect not only the knee flexor muscles strength, but also the ACL-reconstructed knee flexor PT angle. Physical therapists have a wide range of therapeutic modality options to consider in treating patients following ACLR [17, 18] and it is also interesting is whether the knee exercises gradually introduced over time after the reconstruction of ACL by physiotherapists influence the angle at which the PT occurs. Theoretically, exercises performed under the direct supervision of a physiotherapist should be more effective than exercises performed independently by patients who gave up supervised physiotherapy, and continued the therapeutic procedure in the form of gym-based exercises [19]. However, because generally most studies of muscle strength related to ACLR use PT as the primary parameter, the effect of postoperative physiotherapy supervision duration on the PT angle shift towards extension observed in patients after ACLR using STGR tendon autograft has currently not been demonstrated.

This study aimed to assess whether the knee flexor muscles PT angle shift in patients after ACLR using ipsilateral STGR autograft is related to the duration of supervised postoperative physiotherapy.

## Materials and methods

### Participants

The study used an observational cohort study (retrospective study) design. The experiment was approved by the Senate Committee on Ethics of Scientific Research of the Academy of Physical Education in Wrocław, Poland in 2006, and was conducted according to the ethics guidelines and principles of the Declaration of Helsinki. The study was carried out in an academic centre in 2006–2018, and included patients who started the postoperative physiotherapeutic procedure after ACLR in the physiotherapy centre where the study was conducted between 2006 and 2017. All of the patients that participated in the study were informed of the purpose and approach to be used and signed an informed consent form to participate in the study prior to starting the postoperative physiotherapeutic procedure. The participants from the control group were informed of the purpose and approach to be used and signed an informed consent form to participate in the study prior to the performed measurements. The individual in this manuscript has given written informed consent for his photographs to be published in a PLoS Journal.

The initial sample consisted of 230 male patients who started the postoperative physiotherapy after ACLR in the physiotherapy centre where the study was conducted between 2006 and 2017. Because the knee muscle torque is significantly different between males and females (20), the initial sample included only male patients.

The patients were excluded if they had at least one of the following diagnosed medical problems: heart disease, high blood pressure, asthma or pulmonary disease, diabetes (*n* = 1), ulcer or stomach disease, kidney disease, liver disease, anaemia or other blood disease, osteoarthritis, degenerative osteoarthritis, rheumatoid arthritis, back pain, Lyme disease, or alcoholism. Additionally, the following patients were excluded: (1) patients who underwent ACLR with the use of a method other than autologous ipsilateral STGR tendon graft (*n* = 20); (2) participants with any abnormalities in the contralateral knee; (3) participants who underwent at least one of the following procedures: medial and/or lateral meniscectomy, medial and/or lateral meniscal transplant, posterior cruciate ligament repair (*n* = 1), or medial or/and lateral collateral ligament repair/reconstruction; (4) participants with a history of extensor mechanism surgery; (5) patients with a history of osteoarthritis surgery other than shaving (*n* = 5); (6) participants exhibiting one of the following: preoperative activity level, participant in highly competitive sports (*n* = 2), occasional sporting participant preoperatively, and non-sporting participant preoperatively; (6) participants less than 18 years old (n = 8).Fifty patients from the initial sample were lost to follow-up which means that they started physiotherapy after the ACLR, nevertheless they did not report to the measurements at least half a year after the reconstruction.

Finally, 143 male patients were enrolled in the study. Retrospectively, the patients were divided into those who completed supervised physiotherapy (<6 months) followed by an independent return to structured gym exercises and return to activity (Group I; *n* = 77) and patients who completed supervised physiotherapy (≥6 months) and supervised return to sport activity (Group II; *n* = 66).

The mean follow-up was 6.84 ± 1.49 months in Group I and 6.85 ± 1.46 months in Group II (Table 1).

**Table 1:** Characteristics of the studied sample.

	Group I *n* = 77	Group II *n* = 66	Group III *n* = 98	*p*
Age (years)	29.23 ± 7.36	29.44 ± 8.99	27.74 ± 8.42	0.339
Body mass (kg)	82.47 ± 11.31	78.71 ± 10.86	81.70 ± 10.76	0.101
Body height (m)	1.81 ± 0.06	1.81 ± 0.07	1.81 ± 0.06	0.913
Dominant limb right/left (*n*)	68/9	58/8	93/5	n/a
Dominant involved limb (*n*)	38	47	n/a	n/a
Time since ACLR (weeks)	29.75 ± 6.47	29.80 ± 6.34	n/a	0.963
Physiotherapy (weeks)	16.47 ± 6.63	29.95 ± 6.32	n/a	**≤0.001**

Values expressed as the arithmetic mean and standard deviation, ±. ACLR, anterior cruciate ligament reconstruction; Group I, fully supervised postoperative physiotherapy participants; Group II, participants with a shorter duration of postoperative physiotherapy supervision; Group III, control group; *n*, number of individuals in the studied group; n/a, not applicable; *p*, level of significance; supervised physiotherapy, postoperative physiotherapy supervision duration. *p* < 0.050 is indicated in bold.

The results of the patients after ACLR were compared to 98 healthy male volunteers (Group III). The initial sample of male volunteers was 143; however, 45 of them were excluded because of a history of musculoskeletal injuries.

The three studied groups were comparable in terms of age, body mass, and body height. The characteristics of the groups are presented in Table 1.

The leg dominancy in Group I and Group II was not considered in the statistical analysis, as according to the Yoon et al. (1991), there are no differences between dominant and nondominant limbs in terms of knee muscle torque measured under isokinetic conditions [20].

### Surgical procedure (ACLR group)

All the studied patients from the ACLR group underwent post-traumatic primary unilateral one-incision arthroscopically assisted ACLR with the use of autologous ipsilateral double strand semitendinosus–double strand gracilis graft (STGR). The reconstructions were performed by three senior surgeons. In Group I, 71 patients underwent single bundle ACLR, and six patients underwent double-bundle ACLR. In Group II, 48 patients underwent single-bundle, and 18 patients underwent double-bundle ACLR. In all cases, the transtibial technique was utilized. Tourniquet use was the same in all patients. Femoral nerve blocks were not used.

### Postoperative physiotherapy

Supervised physiotherapy means that all physiotherapy sessions were held at the rehabilitation centre where the study was conducted under the strict supervision of a physiotherapist. The physiotherapy was performed by a team of three experienced physiotherapists in accordance with one physiotherapy programme determined in advance [15]. The postoperative physiotherapy followed the procedure described by Czamara (2008) [15] and included four consecutive stages: stage 1, from the day of surgery to the 5^th^ week after the surgery (frequency of visits was 4–5 times a week); stage 2, from the 6^th^ to the 12^th^ week after the surgery (frequency of visits was 4 times a week); stage 3, from 13^th^ week to the 19^th^ week after the surgery (frequency of visits was 4 times a week); and stage 4, from the 20^th^ week to individually over 6–9 months after surgery (frequency of visits was 3 times a week). A single physiotherapeutic visit lasted on average 2 hours. The supervised physiotherapy sessions were regularly monitored [21].

Group I included patients who completed supervised physiotherapy (<6 months) followed by an independent return to structured gym exercises and return to activity. Those patients underwent an average of 3.85 ± 1.53 months of supervised postoperative physiotherapy in the rehabilitation centre where the study was conducted. Therefore, most of them underwent an average of three stages of the procedure, and based on reasons independent of their orthopaedic surgeons and physiotherapists, they refused to continue the supervised physiotherapy. The patients were informed about the main goals and characteristics of the remaining physiotherapeutic stages and independently returned to structured gym exercises and activity. The patients were called after six months after ACLR and asked to participate in the study.

Group II underwent the full four-staged physiotherapeutic procedure in the rehabilitation centre where the study was conducted (≥6 months) that was followed by a supervised return to sport activities. The measurements were a part of a standard test battery performed in the rehabilitation centre where the study was conducted [21].

The duration of postoperative physiotherapy supervision by a physiotherapist was significantly different between the two groups of ACL-reconstructed patients (*p*<0.001); the average was 3.85 ± 1.53 months in Group I and 6.89 ± 1.45 months in Group II (Table 1).

### Procedures

#### Clinical examination

Prior to the PT measurements, all the study participants underwent an examination involving a bilateral manual Lachman test and pivot shift test. The examination was performed by the same orthopaedic surgeon.

#### Peak torque measurements

All the studied participants underwent measurements of knee flexor muscle torque under isokinetic conditions using the Humac Norm Testing & Rehabilitation System (CSMI Computer Sports Medicine, Inc., Stoughton, MA, USA). All the measurements were performed by the same examiner. The measurements in the three studied groups were performed once. The mean time between the ACLR and the measurements in the two groups with ACL-reconstructed patients was comparable; the average duration was 6.84 ± 1.49 months in Group I and 6.85 ± 1.46 months in Group II (Table 1).

The participants were asked to abstain from unaccustomed strenuous exercise for at least 24 hours before the testing and to avoid eating a heavy breakfast in the morning before the test and eating within two hours of the test. The participants were dressed in a comfortable sport outfit and sport shoes.

The measurements were preceded by a 12-minute warm-up on a cycloergometer. The measurements were performed with a participant in the sitting position with 90 degrees of hip flexion. The length of the lever arm was 42 cm for all the participants. The trunk and examined limb were stabilized using belts. The second limb was stabilized with a support. The participant’s arm was crossed on his chest, and his head was leaned on the chair. The foot of the examined limb was dorsiflexed [22].

Gravity correction was used for all tests according to the Computer Sports Medicine, Inc. (CSMi) Humac2009/Norm Application Program User’s Guide. In order to enable gravity correction, the weight of the test limb was measured by Humac before the measurement was started. From this the Humac computed the MaxGet (Maximum Gravity Effected Torque).

The torque measurements were performed bilaterally, starting with the uninvolved limb in Groups I and II and with the dominant limb in Group III. Two series of alternate concentric repetitions for extension and flexion of the knee joint were performed–initially with a constant angular velocity of 180°/s (10 repetitions) and followed by a constant velocity of 60°/s (5 repetitions). There was a 120 s long interval between the two series of repetitions. Each set of repetitions was proceeded by one trial repetition. Between the trial repetitions and the sets of repetition, there was a 10 s long break. The examiner used verbal “start” and “stop” commands [22].

**Fig 1:**
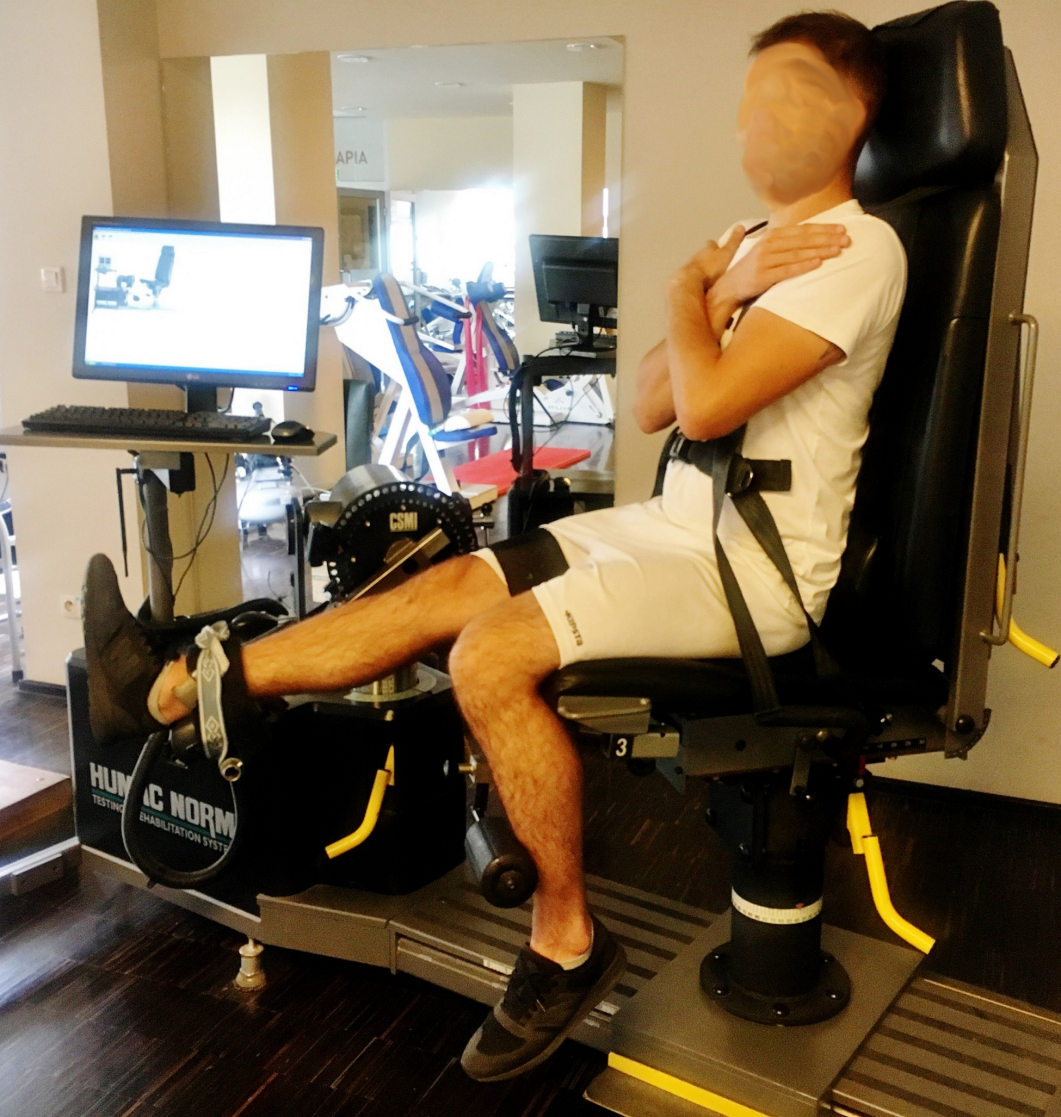
Anatomical zero as the starting position for the knee flexor muscle PT measurements.

**Fig 2:**
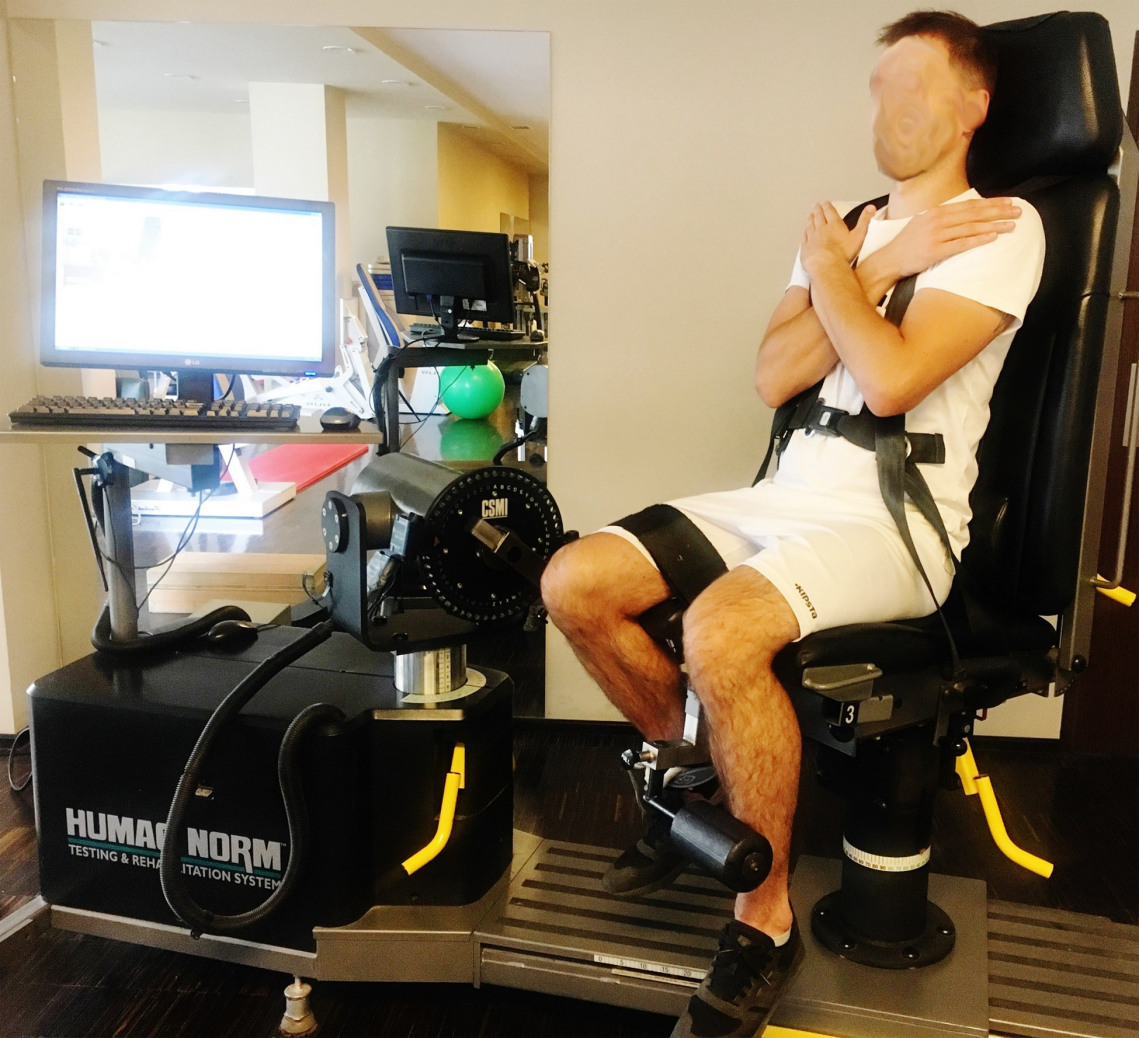
Maximal flexion of the examined limb as the final position for the knee flexor muscle PT measurements.

**Fig 3:**
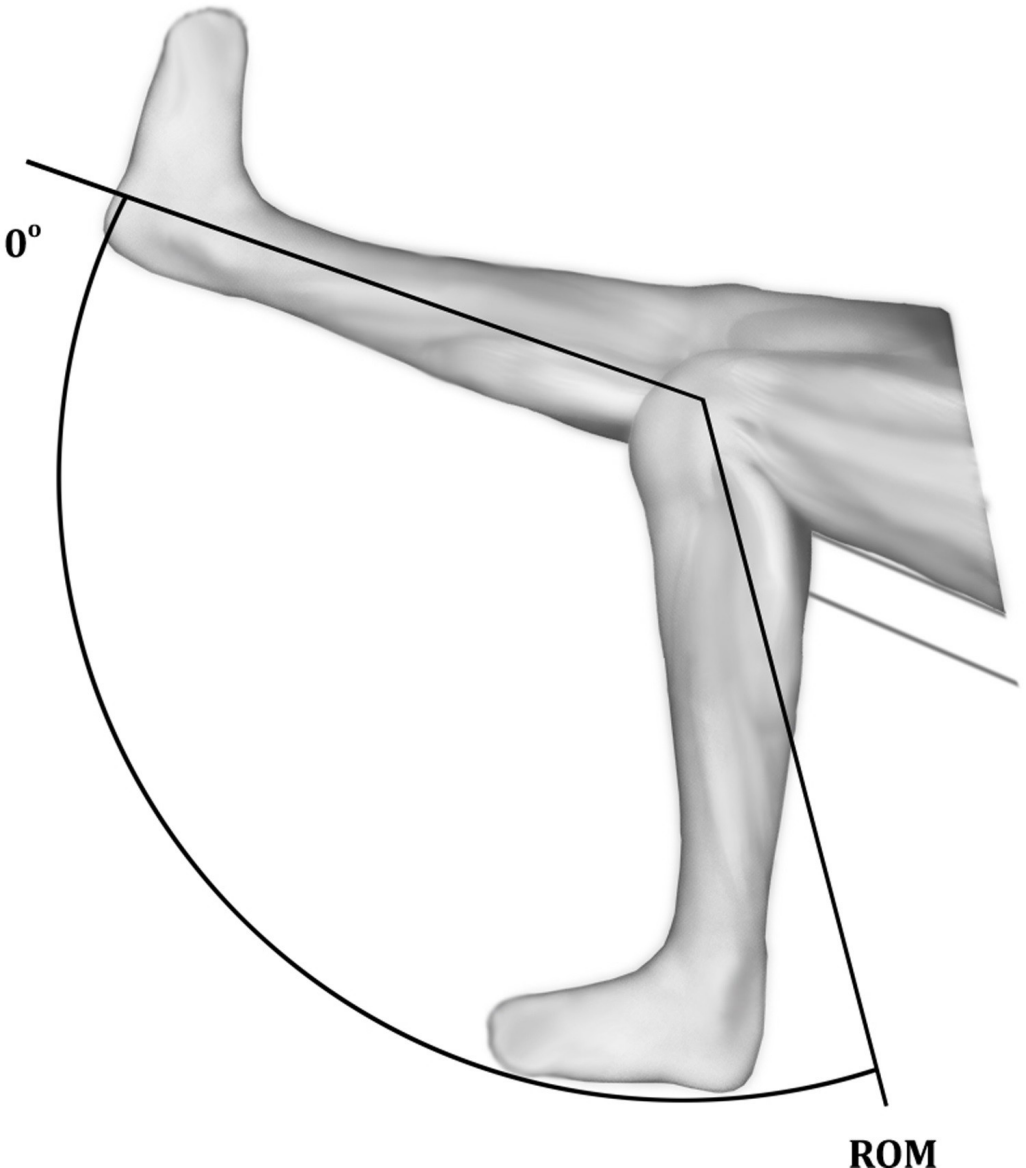
The ROM reflected the range of motion through which knee flexor muscle strength was assessed with dynamometer axis set according to the examined knee axis. The starting position from which the ROM was measured was the position of the extension of zero degrees (no hyperextension).

It is worth noting that in all participants, the zero point was anatomical zero (Fig 1); therefore, the ROM was the angular path from the anatomical zero to the maximum flexion of the knee joint during the measurement (Figs 2 and 3).

The following parameters were included in the analysis: PT in the best repetition at 180°/s, (N*m); PT angle at 180°/s (°); ROM at 180°/s (°). The PT was normalized to body mass and expressed as relative PT, RPT (N*m*kg^-1^). Additionally, the RPT limb symmetry index (LSI) and absolute difference in terms of PT angle and ROM were calculated.

In order to avoid potential so called interviewer bias, we standardized examiner interaction with study participants. The examiner who was responsible for data collection and location of the examined patients in particular groups on the basis of postoperative physiotherapy supervision duration was blinded.

The minimum size of the tested sample was determined based on other studies using the angle of PT of knee joint flexor muscles. According to Yosmaoğlu et al. (2017) [12], who performed a power analysis based on previous studies by Brockett et al. (2004) [23] and Proske et al. (2004) [24], three groups of 28 participants each would be the minimum required to demonstrate a 95% difference in the angle of PT.

### Data analysis and statistics

The LSI for each patient in Group I and Group II was calculated by dividing the result obtained in the involved limb by the result obtained in the uninvolved limb, with the result multiplied by 100. In Group III, the LSI for each participant was calculated by dividing the result obtained in the nondominant limb by the result obtained in the dominant limb, with the result multiplied by 100. The LSI was used to define limb symmetry deficits with values closer to 100 indicating smaller deficits.

The absolute difference between two studied limbs in angle PT and ROM was calculated. The absolute difference of two real numbers *x*, *y* was given by |*x* − *y*|, the absolute value of their difference. The *x* value was the value obtained in the uninvolved limb in Groups I and II and that obtained in the dominant limb in Group III. The *y* value was the consecutively involved limb in Group I and Group II and the nondominant limb in Group III.

IBM SPSS Statistics Subscription, Base Edition Authorized User Per Month (IBM Ireland Product Distribution Limited, IBM House, Dublin, Ireland) and Microsoft Office Excel 365 Personal (Microsoft Corporation, Redmond, WA, USA) were used for the statistical analysis. The arithmetic mean and the standard deviation for the three examined groups were calculated for age, body mass, body height, time since ACLR and the measurements, postoperative physiotherapy supervision duration, RPT, PT angle, ROM, LSI, and between-limb absolute difference value of PT angle and ROM. The normality Shapiro-Wilk test was used to study the distribution of the variables. Depending on the result of the normality test, a parametric or non-parametric test for dependent samples was performed. The intra-group comparison refers to the comparison between the involved and uninvolved limbs in Groups I and II and between the dominant and nondominant limbs in Group III. One-way ANOVA and post hoc Bonferroni tests were used in the inter-group comparisons between the involved limb in Group I, the involved limb in Group II, the dominant limb in Group III or between the uninvolved limb in Group I, uninvolved limb in Group II, and nondominant limb in Group III. The linear Pearson’s correlation coefficient calculation was also performed. Correlation coefficient *r*-value was calculated for the strength and direction of a linear relationship between the duration of supervised postoperative physiotherapy in ACL-reconstructed patients (combined Group I with Group II) with the selected parameters. The magnitude of all bivariate associations were classified as negligible (0.0–0.3), low (0.31–0.5), moderate (0.51–0.7), high (0.71–0.9), and very high (0.9–1.0) [25]. Statistical significance was set at *p*<0.050.

## Results

### Clinical examination

Analysis of the manual anterior tibial translation testing results based on the Lachman test revealed no abnormalities in any of the studied groups. The pivot shift was negative in all studied participants.

### Relative peak torque

The mean RPT values obtained at 180°/s were significantly lower in the involved limb compared to the uninvolved limb in Group I (Table 2). The mean RPT values obtained at 180°/s were comparable in the involved and uninvolved limbs in Group II and in the dominant and nondominant limbs in Group III (Table 2).

**Table 2:** Side-to-stide comparison within the three studied groups in terms of analysed parameters.

Knee flexor muscles at 180°/s					
Parameters	Limb	Group I	Group II	Limb	Group III
RPT (N*m*kg^-1^)	Involved	0.98 ± 0.26	1.16 ± 0.24	Dominant	1.09 ± 0.24
	Uninvolved	1.13 ± 0.26	1.18 ± 0.24	Nondominant	1.09 ± 0.23
	*p*	**≤ 0.001**	0.055	*p*	0.815
PT angle (°)	Involved	45.97 ± 9.70	45.06 ± 8.99	Dominant	52.33 ± 10.06
	Uninvolved	55.45 ± 13.45	50.88 ± 10.84	Nondominant	50.89 ± 10.40
	*p*	**≤ 0.001**	**≤ 0.001**	*p*	0.161
ROM (°)	Involved	108.90 ± 10.45	110.63 ± 9.07	Dominant	110.36 ± 8.21
	Uninvolved	112.46 ± 16.38	113.41 ± 8.28	Nondominant	111.59 ± 8.42
	*p*	**0.029**	**≤ 0.001**	*p*	0.073

Values expressed as the arithmetic mean and standard deviation, ±. Group I, fully supervised postoperative physiotherapy participants; Group II, participants with a shorter duration of postoperative physiotherapy supervision; Group III, control group; *p*, level of significance; PT, peak torque; RPT, relative peak torque; ROM, range of motion. *p* < 0.050 is indicated in bold.

The mean RPT value at 180°/s was significantly lower in the involved limb in Group I than in the involved limb in Group II and dominant limb in Group III (Table 3). The values of RPT at 180°/s were comparable in the involved limb in Group II and dominant limb in Group III. The RPT values at 180°/s were comparable in the uninvolved limbs in Group I and Group II and in the nondominant limb in Group III (Table 3).

**Table 3:** The between-groups comparison in terms of analysed parameters.

Knee flexor muscles at 180°/s				
Studied limbs	Involved (Group I) vs involved (Group II) vs dominant (Group III)		Uninvolved (Group I) vs uninvolved (Group II) vs nondominant (Group III)	
Parameters	Anova (*p*)	Bonferroni post hoc test	Anova (*p*)	Bonferroni post hoc test
RPT (N*m*kg^-1^)	**≤0.001**	Group I vs Group II, ***p* ≤ 0.001**; Group I vs Group III, ***p* = 0.011**; Group II vs Group III, *p* = 0.223	0.062	n/a
PT angle (°)	**≤ 0.001**	Group I vs Group II, *p* = 1.000; Group I vs Group III, ***p* ≤ 0.001**; Group II vs Group III, ***p* ≤ 0.001**	**0.018**	Group I vs Group II, *p* = 0.058; Group I vs Group III, ***p* = 0.031**; Group II vs Group III, *p =* 1.000
ROM (°)	0.459	n/a	0.611	n/a

Group I, fully supervised physiotherapy participants; Group II, participants with a shorter duration of physiotherapy supervision; Group III, control group; n/a, not applicable; *p*, level of significance; PT, peak torque; RPT, relative peak torque; ROM, range of motion. *p* < 0.050 is indicated in bold.

The knee flexor muscles RPT LSI at 180°/s was significantly worse in Group I in comparison to Group II, and Group III (Table 4). There were no significant differences between Group II and Group III.

**Table 4:** The side-to-side comparison within the three studied groups in terms of limb symmetry index and absolute difference.

Knee flexor muscles at 180°/s						
Parameters		Group I	Group II	Group III	Anova (*p*)	Bonferroni post-hoc test
Limb Symmetry Index	RPT	86.74 ± 12.72	98.55 ± 6.19	100.41 ± 8.99	**≤ 0.001**	Group I vs Group II, ***p* ≤ 0.001**; Group I vs Group III, ***p* ≤ 0.001**; Group II vs Group III, *p* = 0.458
Absolute difference (°)	PT angle	11.61 ± 10.85	9.58 ± 6.87	7.95 ± 6.33	**0.014**	Group I vs Group II, *p* = 0.695; Group I vs Group III, ***p* = 0.011**; Group II vs Group III, *p* = 0.637
	ROM	8.23 ± 11.85	5.26 ± 4.32	5.23 ± 4.41	**0.019**	Group I vs Group II, *p* = 0.063; Group I vs Group III, ***p* = 0.031**; Group II vs Group III, *p* = 1.000

Values expressed as the arithmetic mean and standard deviation, ±. Group I, fully supervised postoperative physiotherapy participants; Group II, participants with a shorter duration of postoperative physiotherapy supervision; Group III, control group; *p*, level of significance; PT, peak torque; RPT, relative peak torque; ROM, range of motion. *p*<0.050 is indicated in bold.

### Peak torque angle

The intra-group comparative analysis revealed that an ACL-reconstructed limb knee flexor muscles PT angle at 180°/s was significantly shifted towards extension in comparison to the uninvolved limb in Group I and Group II (Table 2). There were no differences between the dominant and nondominant limbs in Group III (Table 2).

PT angle at 180°/s was significantly greater in Group III compared to the involved limb in Group I and to the involved limb in Group II. The results in the involved limb in Group I and Group II were comparable (Table 3).

The absolute difference in PT angle value at 180°/s was significantly greater in Group I compared to Group III (Table 4). However, the absolute difference was comparable between Group I and Group II and between Group II and Group III.

### Range of motion

The intra-group comparison revealed that the ROM at 180°/s was significantly smaller in the involved limb compared to that in the uninvolved limb in Group I and Group II (Table 2). The ROM did not significantly differ between the dominant and nondominant limb in Group III (Table 2).

In contrast, the inter-group comparison revealed no significant differences between the studied groups (Table 3).

The ROM absolute value differed between groups (Table 4). The absolute difference in ROM was significantly larger in Group I compared to that in Group III. The results were comparable in Group I and Group II, as well as Group II and Group III.

### Correlation with the duration of supervised postoperative physiotherapy

**Fig 4:**
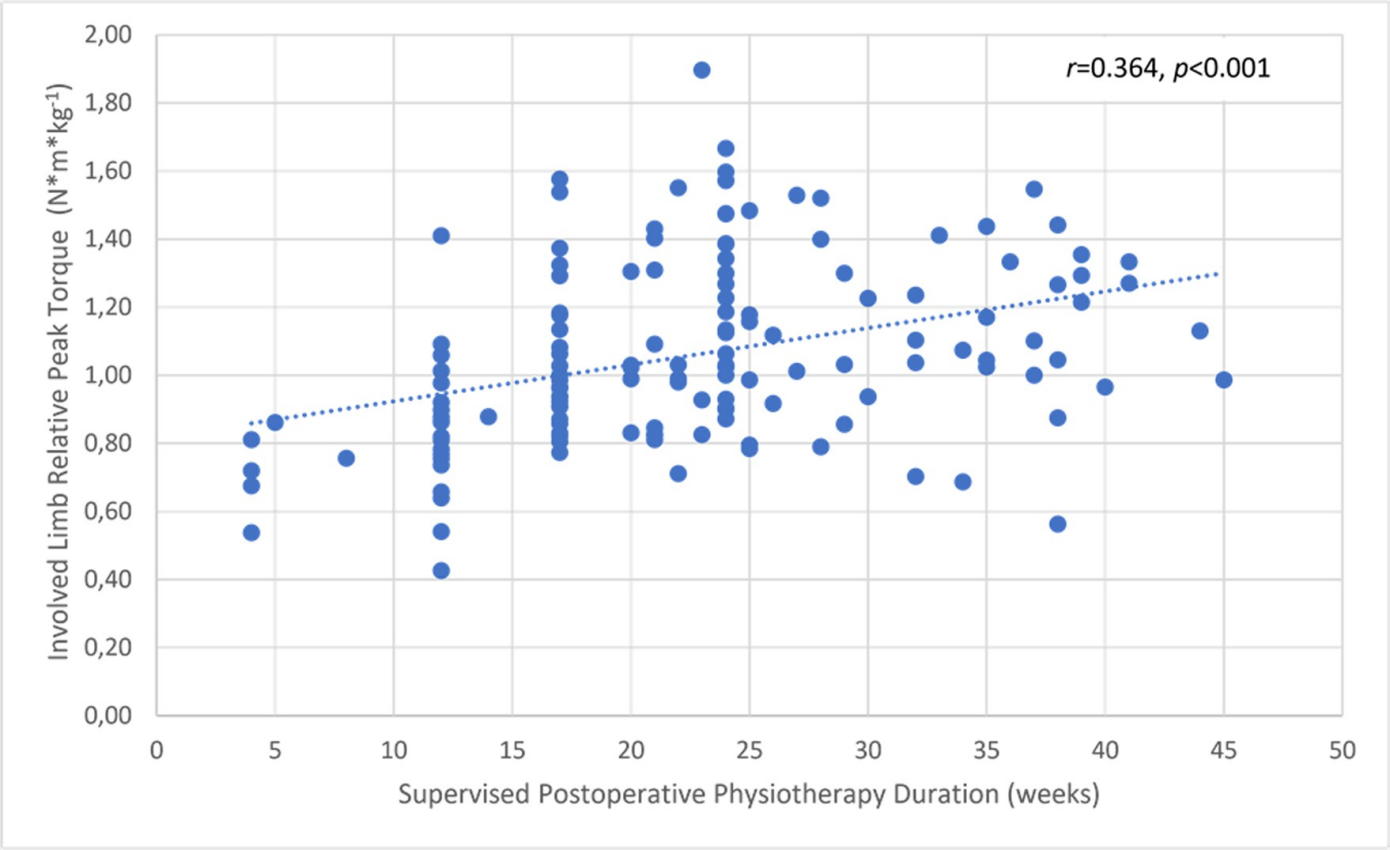
A significant but small positive correlation indicated that higher involved limb knee flexor muscle relative PT value at 180°/s in ACL-reconstructed patients (Group I and Group II, *n* = 143) is associated with longer duration of supervised postoperative physiotherapy.

**Fig 5:**
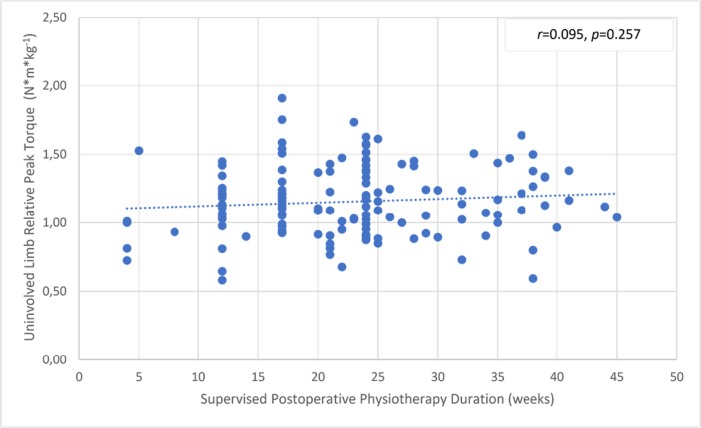
Lack of association of uninvolved limb knee flexor muscle relative PT value at 180°/s in ACL-reconstructed patients (Group I and Group II, *n* = 143) with duration of supervised postoperative physiotherapy.

**Fig 6:**
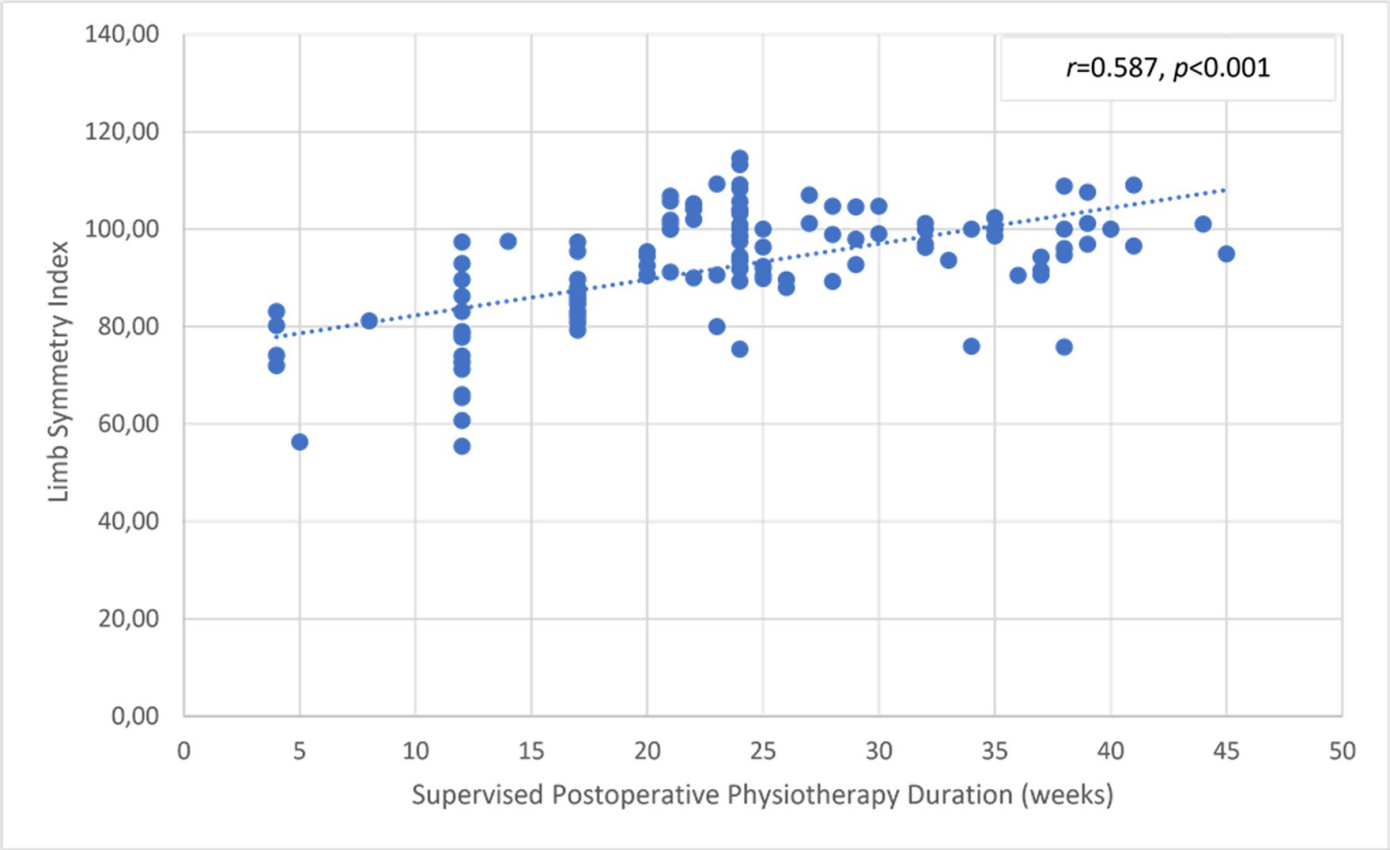
Significant positive moderate correlation indicating that higher knee flexor muscle LSI value at 180°/s in ACL-reconstructed patients (Group I and Group II, *n* = 143) is associated with longer duration of supervised postoperative physiotherapy.

**Fig 7:**
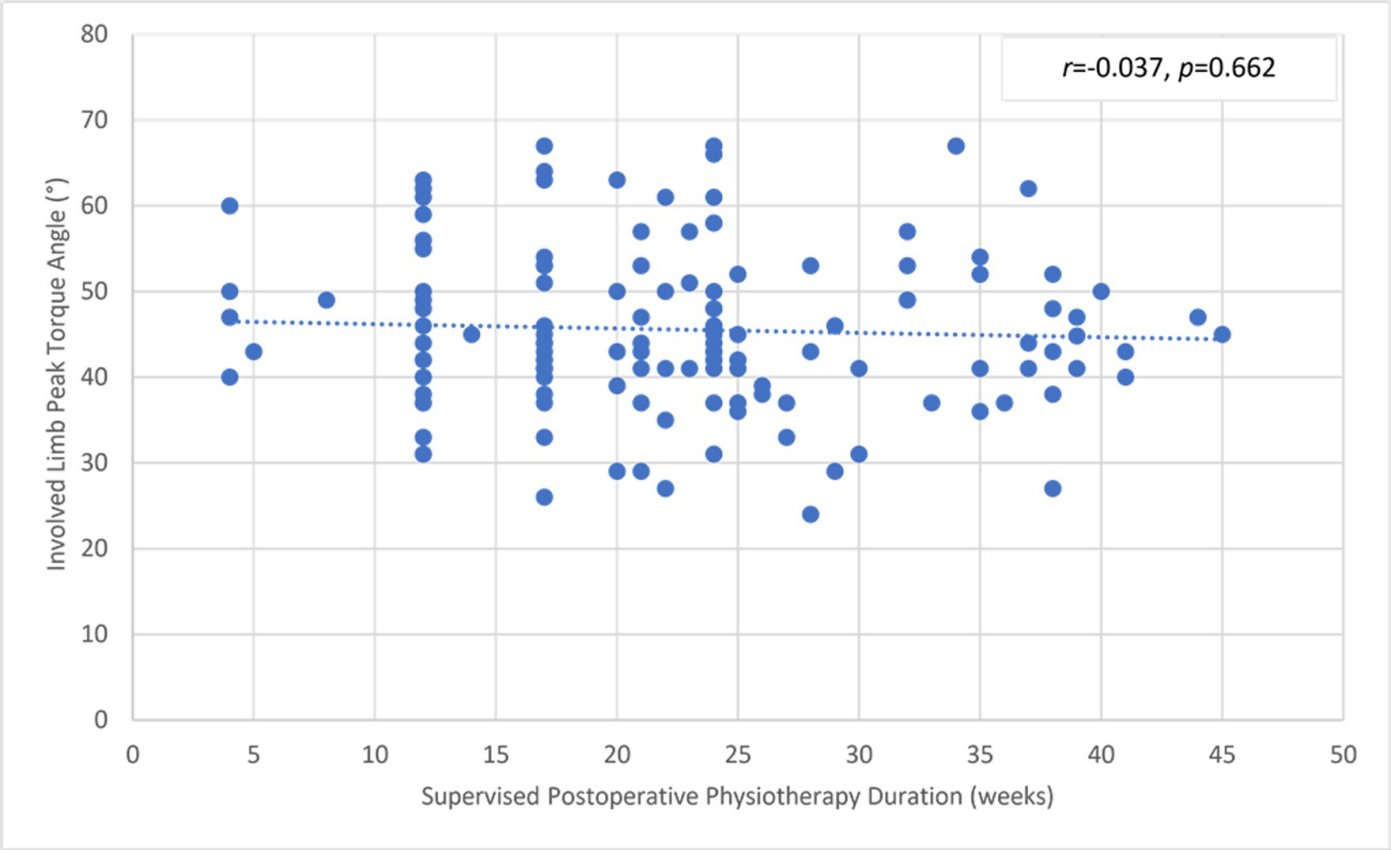
Lack of association of involved limb knee flexor muscle PT angle value at 180°/s in ACL-reconstructed patients (Group I and Group II, *n* = 143) with duration of supervised postoperative physiotherapy.

**Fig 8:**
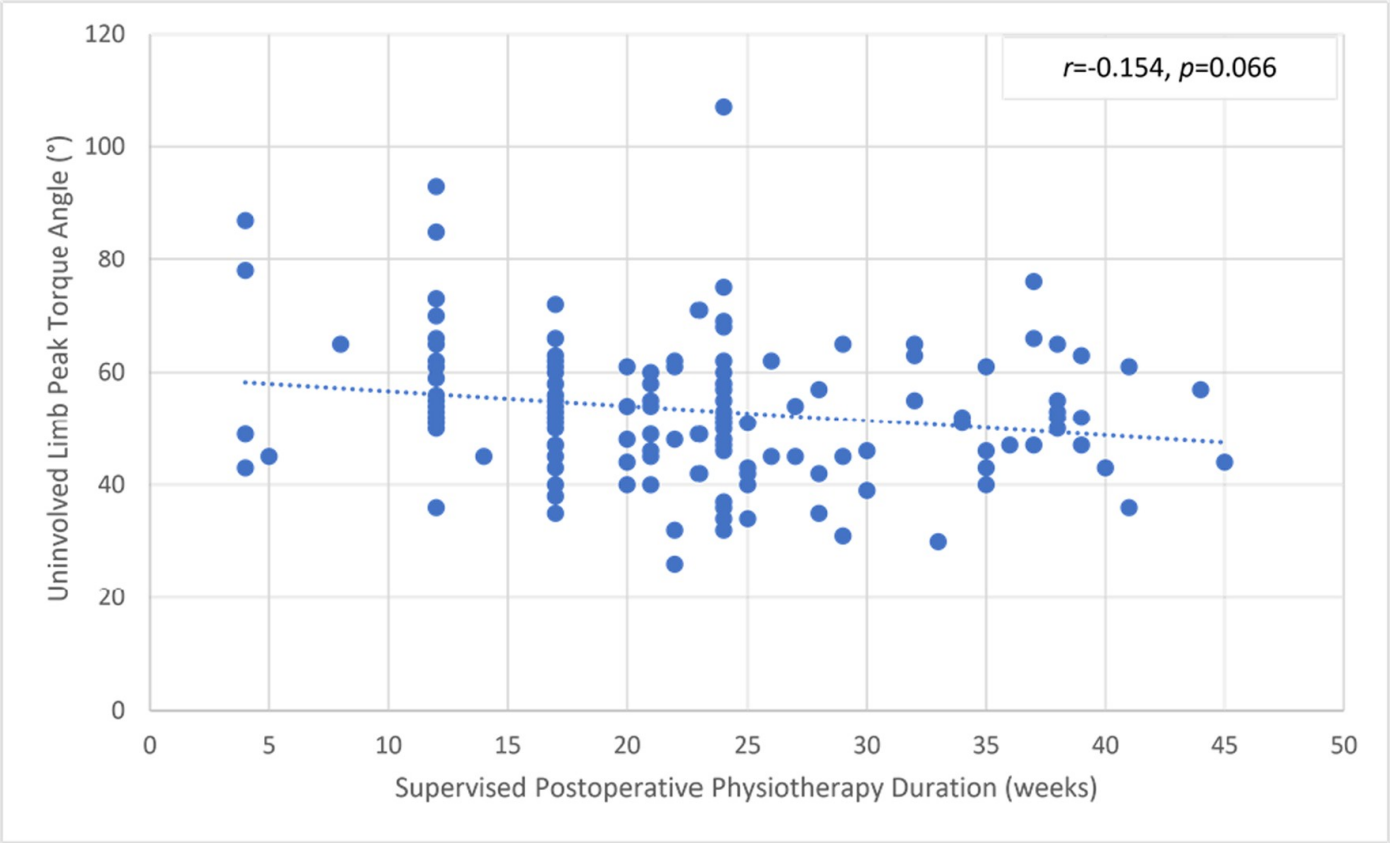
Lack of association of uninvolved limb knee flexor muscle PT angle value at 180°/s in ACL-reconstructed patients (Group I and Group II, *n* = 143) with duration of supervised postoperative physiotherapy.

**Fig 9:**
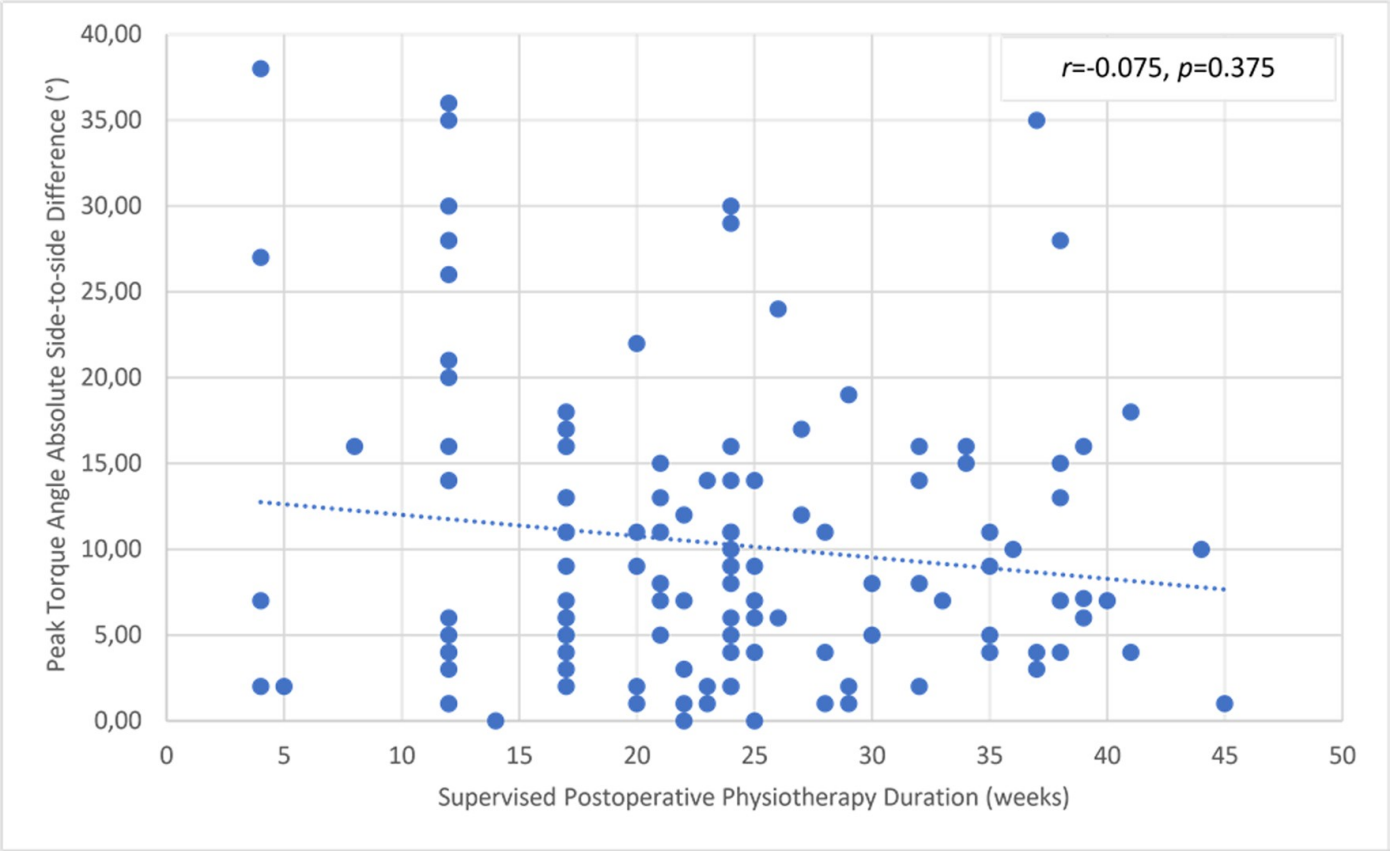
Lack of association of knee flexor muscle PT angle absolute side-to-side difference value at 180°/s in ACL-reconstructed patients (Group I and Group II, *n* = 143) with duration of supervised postoperative physiotherapy.

**Fig 10:**
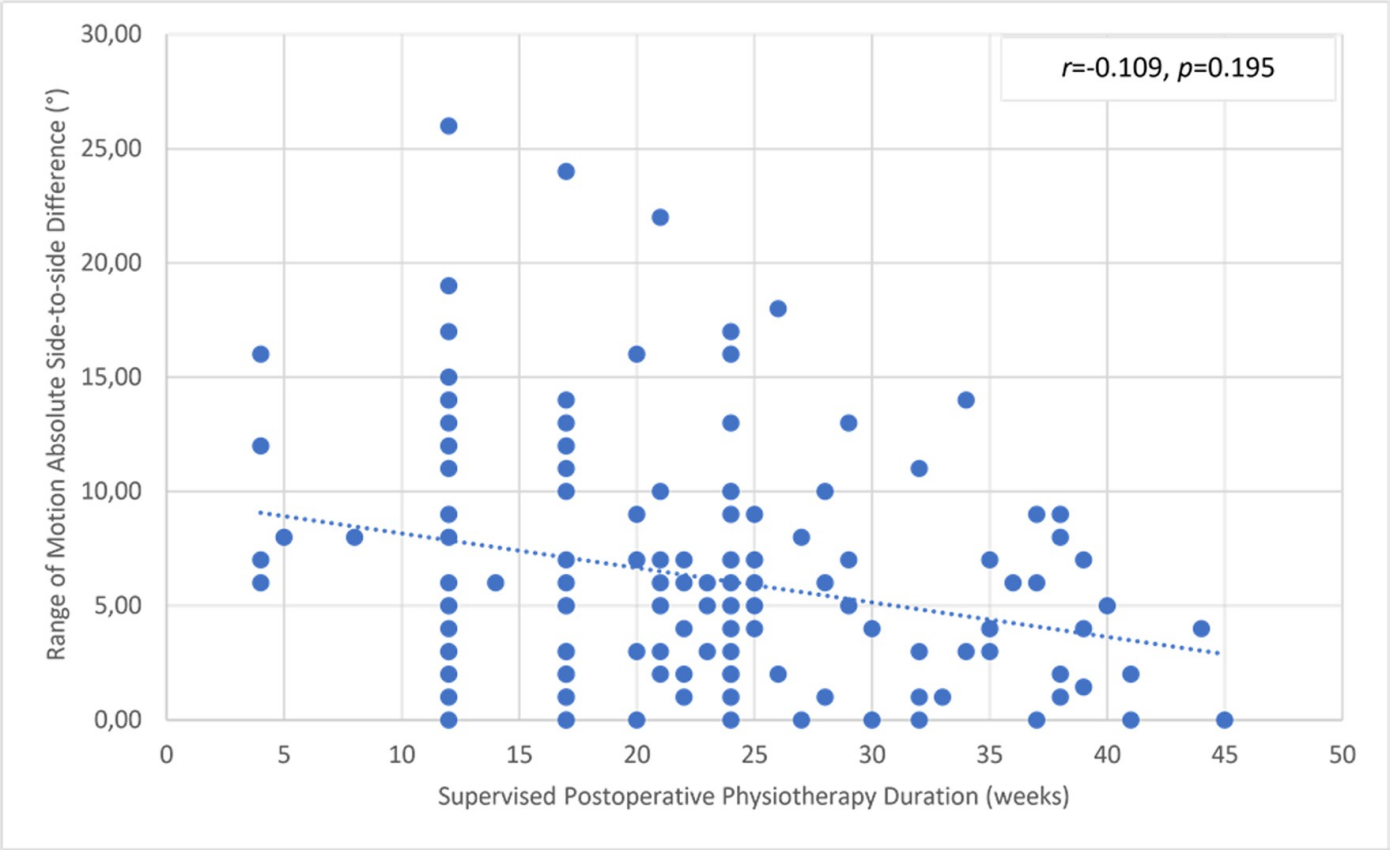
Lack of association of knee flexor muscle ROM absolute side-to-side difference value at 180°/s in ACL-reconstructed patients (Group I and Group II, *n* = 143) with duration of supervised postoperative physiotherapy.

There was a significant but small correlation between the involved knee flexor muscle RPT at 180° and the duration of supervised postoperative physiotherapy (Fig 4). The uninvolved limb RPT value was not correlated with the physiotherapy (Fig 5). The knee flexor LSI was moderately associated with the duration of supervised physiotherapy (Fig 6). There were no correlations between the duration of supervision and PT angle at 180°/s in the involved (Fig 7) and uninvolved limbs (Fig 8), the absolute difference in PT angle (Fig 9), and ROM (Fig 10).

### Knee flexor muscles relative peak torque limb symmetry index distribution

**Fig 11:**
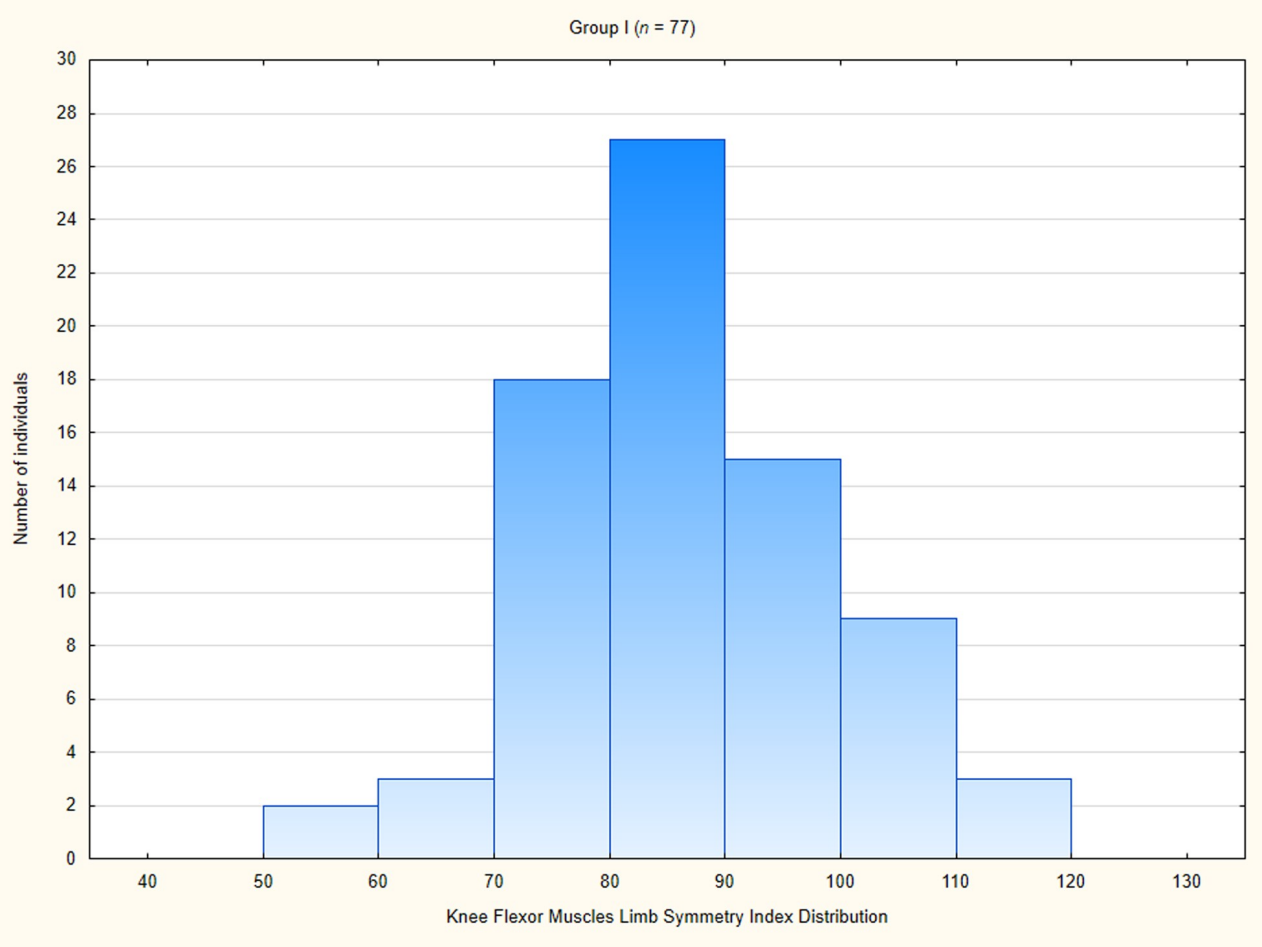
Knee flexor muscle limb symmetry index distribution among patients in Group I.

The knee flexor muscles RPT LSI in Group I amounted averagely 86.74 ± 12.72 (Table 4). The LSI in this group ranged from 55 to 119. In a great majority of patients in the Group I the LSI was smaller than 90 (Fig 11).

**Fig 12:**
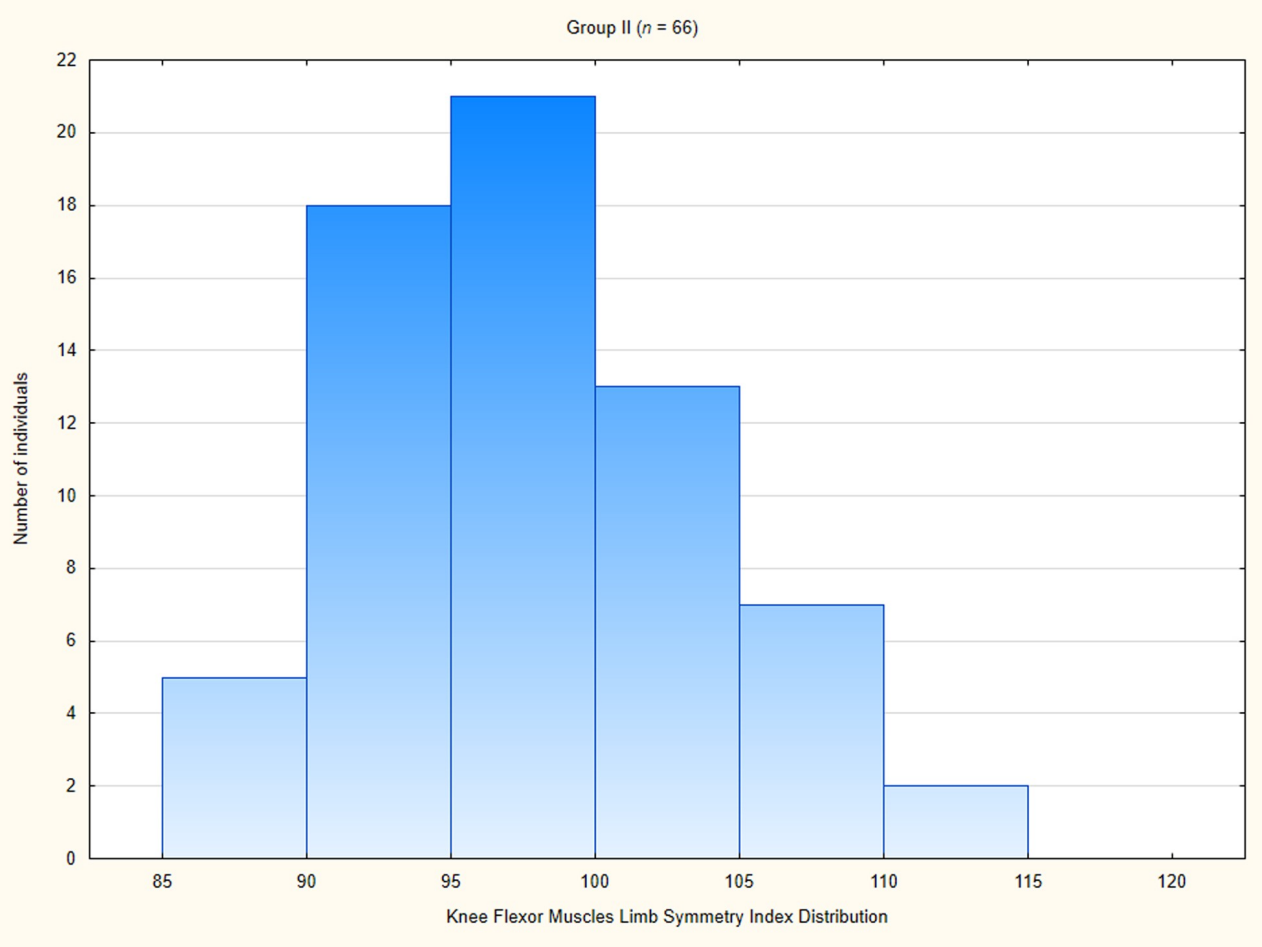
Knee flexor muscle limb symmetry index distribution among patients in Group I.

When it comes to the Group II, the knee flexor muscles RPT LSI amounted averagely 98.55 ± 6.19 (Table 4), ranging from 88 to 115. In a great majority of patients in the Group II the LSI was equal or larger than 90 (Fig 12).

**Fig 13:**
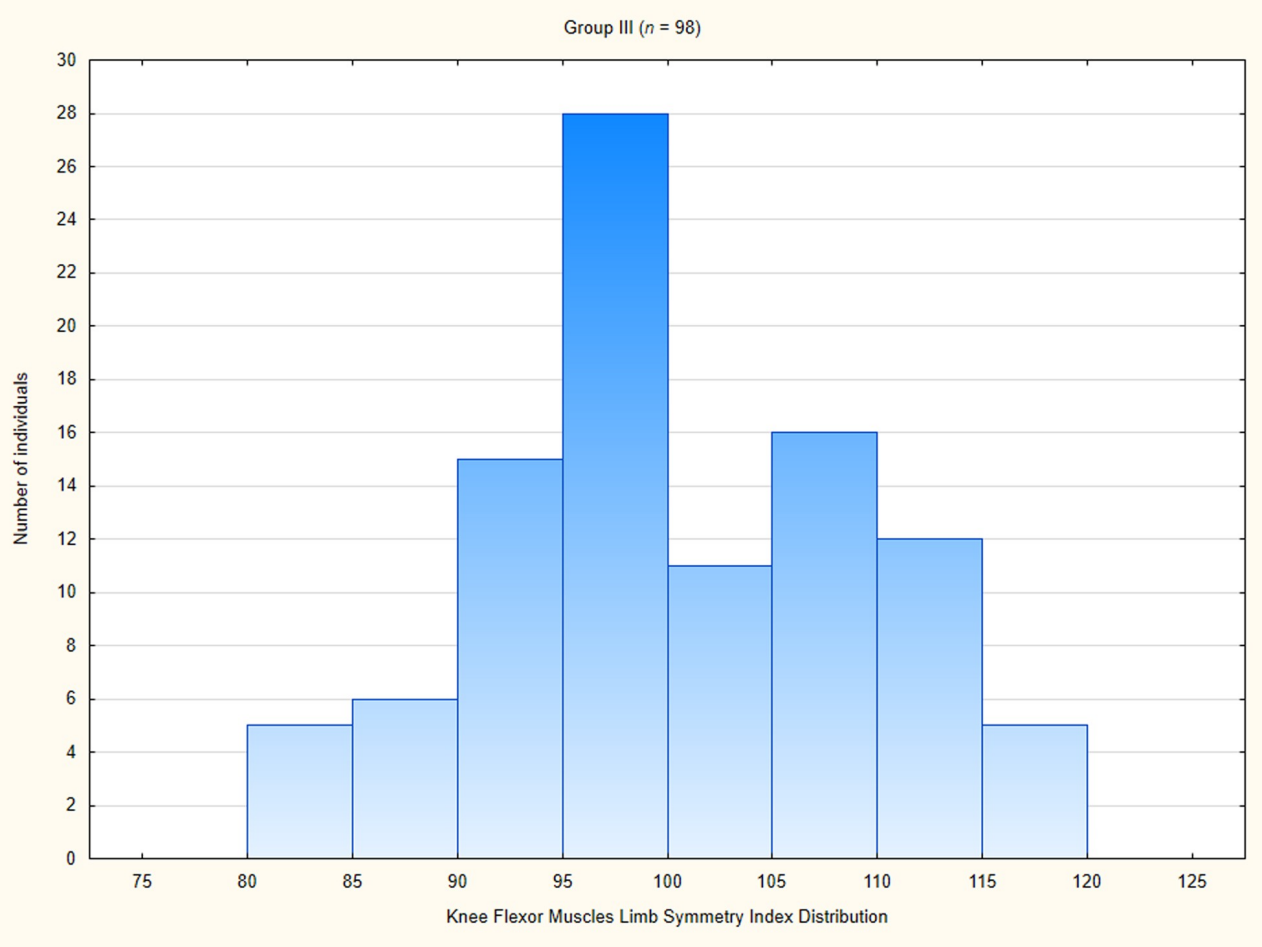
Knee flexor muscle limb symmetry index distribution among participants in Group III.

The knee flexor muscles RPT LSI in Group III amounted averagely 100.41 ± 8.99 (Table 4). The LSI in this group ranged from 80 to 118. In a great majority of participants in the Group III the LSI was equal or larger than 90 (Fig 13).

## Discussion

The main finding of the study was the observation of a shift towards extension of the knee flexor muscle PT angle at 180°/s in males at seven months after ACLR utilizing STGR autograft in the involved limb regardless of the duration of postoperative supervised physiotherapy. The shift towards extension was observed in comparing the ACL-reconstructed limb to the uninvolved limb and in comparing to healthy individuals. The RPT of knee flexor muscles in the involved limb was significantly higher in the group of patients with postoperative physiotherapy supervision duration ≥ 6 months than in patients with supervision duration < 6 months. For the entire sample of ACL-reconstructed patients, there was a significant but small correlation indicating that higher RPT of knee flexor muscles in the involved limb is associated with longer duration of postoperative physiotherapy supervision. Additionally, our analysis revealed that there was a significant moderate association between duration of postoperative physiotherapy supervision and knee flexor muscle LSI. Longer duration of physiotherapy was associated with higher LSI. In patients after ACLR with the use of ipsilateral STGR autograft exercises performed under the direct supervision of a physiotherapist are more effective than exercises performed independently by patients who gave up supervised physiotherapy, and continued the therapeutic procedure in the form of gym-based exercises in terms of the quantitative parameter, which is the PT, nevertheless they do not affect the qualitative parameter which is the angle at which the PT occurs. What’s more, the standard exercises introduced by physiotherapists in patients after ACLR with the use of STGR are insufficient to restore the involved knee flexor muscles PT angle on the level of uninvolved limb and healthy individuals.

The measurement of muscle strength is essential in diagnosing muscle weakness, assessing specific injuries, and evaluating the effectiveness of a physiotherapeutic procedure. The exercise under isokinetic conditions involves a dynamic pre-set fixed velocity, with resistance varying exactly in response to the force applied by the individual, throughout a specified ROM. PT is the most commonly utilized strength variable in isokinetic testing; however, there have been authors who analysed changes in the PT angle in patients after ACLR [12]. According to Yosmaoğlu et al. (2017), the ACL-reconstructed knee flexor muscle PT angle at 180°/s shifts towards extension if the graft is harvested from hamstring tendons [12]. The present study confirmed this observation. We found significant differences in the ACL-reconstructed limbs compared to the uninvolved limbs. The PT angle in ACL-reconstructed limbs was also significantly smaller than that in healthy controls. Interestingly, it was difficult to find any studies comparing the ACL-reconstructed knee flexor muscle PT angle shift towards extension at 180°/s in heathy controls.

It has been known since the mid-1980s that with increasing test velocity, PT occurs later in ROM, indicating that the limb may not have an optimal joint position for muscular performance at high angular velocities and therefore, the recorded PT may not represent the individual’s maximum torque-producing capacity [26]. The decrease in deep ACL-reconstructed knee flexion torque could be due to the atrophy and shortening of the ST after its tendon has been harvested, as well as the lack of compensation from the semimembranosus and biceps femoris due to the architectural differences between the ST and the semimembranosus and biceps femoris [27]. After harvesting the ST for the reconstruction, the biceps femoris and semimembranosus are left as the primary knee flexors; however, they are insufficient to produce torque, especially at deeper flexion angles. According to Nomura et al. (2015), due to the correlation between tendon regeneration, ST muscle shortening, and ST muscle atrophy with decreased knee flexion torque, preserving the morphology of the ST muscle-tendon complex is a very important issue [28]. According to Kannus and Jarvinen (1990), PT tends to occur later in weaker muscles, probably because of slow neural recruitment [29]. This finding is consistent with the assumption that another factor that may be responsible for shifting the PT angle in the ACL-reconstructed knee is an impairment in the neuromuscular modulation system, as the knee joint mechanoreceptor system is disturbed after ACL injury, and the impairment remains even after reconstruction.

Some findings suggest that GR harvesting is determinantal to knee flexor weakness at deep flexion angles and support the use of ST alone [2, 3, 5, 6], even though much of the literature is contradictory in this matter [5, 6, 10]. It is hypothesized that the GR muscles may act to reinforce the action of hamstrings in deep knee flexion due to biomechanical alternation [30]. In response to the ST harvesting, the GR may undergo compensatory hypertrophy, facilitating more anatomic ST regeneration [30]. However, due to the lack of patients after ACLR with ST graft usage, we are unable to support or reject the theory that ST harvest alone is preferable.

The present study confirms the positive role of postoperative physiotherapy in muscle strength in patients after ACLR [31]. The physiotherapy programme followed the procedures described by Czamara (2008) [15]. The first stage aimed to reduce pain and effusion and to restore ACL-reconstructed knee range of motion and gait. The aim of the second stage was to improve the gait pattern and to stimulate proprioception. The third stage was based on the reduction of limb strength asymmetries and teaching proper landing technique and involved running for general endurance training. The fourth stage was generally focused on complex movement pattern exercises, strength, power and specific endurance training [15]. The first postoperative knee flexor muscles exercises in the ACL-reconstructed limb were already introduced on the 1st day after surgery in the form of isometric tensions. The first active knee flexor muscle exercises were introduced in the 5th week postoperatively. Active knee flexor muscle exercises with manual resistance were introduced in the 6th week. The first squats were introduced in the 6th week. Initially, they were performed in a limited range of motion up to 60 degrees of flexion, followed by gradually increasing range of motion. Active knee flexor muscle exercises with gradually increased resistance using the exercise equipment were introduced in the 7th week postoperatively. Exercises under isokinetic conditions were introduced in the 16th week postoperatively. The exercises were first performed in a limited range of motion, and the range of movement was gradually increased. Hamstring stretches in the involved limb were introduced on the 2nd day after surgery [15]. Muscle strength was regularly monitored by physiotherapists [14, 21]. The four-stage physiotherapeutic procedure that lasted at least 6 months resulted in significant improvement in knee flexor muscle RPT values in healthy individuals. The presence of greater knee flexor muscle weakness in the ACL-reconstructed limbs in patients who completed the supervised physiotherapeutic procedure before 6 months postoperatively seems to confirm that the strategy based on structured gym exercises does not fully restore strength in fully supervised patients and healthy individuals. Longer duration of supervised physiotherapy appears to be sufficient to restore knee flexor muscle strength. Knee flexor muscle strength deficits that are present when patients return to sport activities may be hazardous because this muscle group restrains anterior tibial translation, a known contributor to ACL injury [32].

The duration of supervised postoperative physiotherapy was directly correlated with knee flexor muscle LSI. The lower LSI was below the recommended criterion (≥90) for most patients in Group I. It is clear that many patients should have undertaken the supervised physiotherapy required to address postoperative strength deficits. Some patients did obtain the recommended LSI; nevertheless, this variation among patients demonstrates the need to individualize and monitor the progress of rehabilitation. In Group II and Group III, most participants had LSI ≥ 90. Of course, caution is warranted in the use of LSI because it can mask bilateral deficits as the uninvolved limb can also be affected, and may also be experiencing strength weakness after reconstruction [33–35]. The issue of shifting the PT angle towards extension in patients after ACLR utilizing hamstring graft remains unsolved, and in the future, research on the possibility of restoration of the PT angle with specific exercises should be intensified. There exists a strong need for research on specific exercises aimed at restoring the PT angle of muscles from which the tendon was harvested for the reconstruction.

The main limitation of the study was the lack of the output data of the examined ACL-reconstructed patients from the period before reconstruction. Therefore, we applied very strict initial sample exclusion criteria, and the results of the ACL-reconstructed patients were compared to healthy individuals matched in terms of gender, age and general body composition. An additional limitation was the uneven number of participants between the three studied groups; however, it did not affect the side-to-side comparison results, which were the main findings of the study. The manual ligament laxity assessment may also be considered as a limitation. What’s more, it should be highlighted, that the study was conducted only on male participants, thus results cannot be generalized to female population. The clinical and functional importance of the knee muscle flexor PT angle shift towards extension in patients after ACLR with autologous ipsilateral hamstring graft is still an unresolved issue. There also remains a need for resolving the conflict regarding the repeatability of the PT angle measurement [36], whereas the reproducibility of isokinetic measurements is considered to be very high. In the future, potential gender-specific differences in terms of strength deficits in deeper angles of knee flexion should be investigated.

## Conclusions

The knee flexor muscle PT angle shift towards extension at 180°/s in males at seven months after ACLR utilizing STGR ipsilateral autograft was observed in the involved limb regardless of the duration of supervised postoperative physiotherapy. The shift towards extension was observed in comparing the ACL-reconstructed limb to the uninvolved limb and in comparing to the healthy individuals. The knee flexor muscles RPT in the involved limb was significantly higher in the group of patients with postoperative physiotherapy supervision duration ≥ 6 months, than in patients with supervision duration < 6 months. For the entire sample of ACL-reconstructed patients, there was a significant but small positive correlation between the ACL-reconstructed knee flexor muscle RPT and longer duration of supervised postoperative physiotherapy. Additionally, there was a significant moderate association between duration of supervised postoperative physiotherapy and knee flexor muscle LSI. The longer the duration of physiotherapy supervision, the higher the LSI. The present study may justify the superiority of fully supervised postoperative physiotherapy after ACLR over unsupervised therapy in terms of RPT LSI restoration.

## Supporting information

S1 STROBE checklist (DOCX)

## Abbreviations

ACL: anterior cruciate ligament
ACLR: anterior cruciate ligament reconstruction
GR: gracilis
PT: peak torque
ROM: range of motion
RPT: relative peak torque
ST: semitendinosus
STGR: semitendinosus and gracilis
